# The Risk of Para-Aortic Lymph Node Metastases in Apparent Early Stage Ovarian Cancer

**DOI:** 10.3390/medicina56030108

**Published:** 2020-03-03

**Authors:** Nicolae Bacalbasa, Irina Balescu, Mihaela Vilcu, Simona Dima, Camelia Diaconu, Laura Iliescu, Alexandru Filipescu, Mihai Dimitriu, Iulian Brezean

**Affiliations:** 1 “Carol Davila” University of Medicine and Pharmacy, 020021 Bucharest, Romania; nicolae_bacalbasa@yahoo.ro (N.B.); mihaela.vilcu@gmail.ro (M.V.); drcameliadiaconu@gmail.com (C.D.); laura.iliescu@gmail.ro (L.I.); alexandru.filipescu@gmail.ro (A.F.); mihai.dimitriu@gmail.ro (M.D.); iulian.brezean@gmail.ro (I.B.); 2 Department of Obstetrics and Gynecology, “I. Cantacuzino” Clinical Hospital, 030167 Bucharest, Romania; 3 Department of Visceral Surgery, Center of Excellence in Translational Medicine, “Fundeni” Clinical Institute, 022328 Bucharest, Romania; simonadima@gmail.ro; 4 Department of Surgery, “Ponderas” Academic Hospital, 021188 Bucharest, Romania; 5 Department of Visceral Surgery, “I. Cantacuzino” Clinical Hospital, 030167 Bucharest, Romania; 6 Department of Internal Medicine, Clinical Emergency Hospital of Bucharest, 105402 Bucharest, Romania; 7 Department of Internal Medicine, “Fundeni” Clinical Institute, 022328 Bucharest, Romania; 8 Department of Obstetrics and Gynecology, “Elias” Emergency University Hospital, 011461 Bucharest, Romania; 9 Department of Obstetrics and Gynecology, “St Pantelimon” Emergency Hospital, 021661 Bucharest, Romania

**Keywords:** early stage, ovarian cancer, para-aortic lymph node metastases

## Abstract

*Background and objectives:* To identify the risk factors for para-aortic lymph node metastases in cases with presumed early stage ovarian cancer. *Materials and methods*: Between 2014 and 2019, 48 patients with apparent early stage ovarian cancer were submitted to surgery. In all cases, pelvic and para-aortic lymph node dissection was performed for staging purposes. *Results*: Among the 48 cases we identified nine cases with positive pelvic lymph nodes and 11 cases with positive para-aortic lymph nodes. The positivity of the retrieved lymph nodes was significantly correlated with the histopathological subtype represented by serous histology (*p* = 0.02), as well as with the degree of differentiation (*p* = 0.004). *Conclusions*: Patients with serous ovarian carcinomas in association with a poorer degree of differentiation are at risk of associated lymph node metastases even in presumed early stages of the disease. Therefore, lymph node dissection should be performed in such cases in order to provide adequate staging and tailoring of further treatment.

## 1. Introduction

Ovarian cancer represents a common malignancy affecting women worldwide that unfortunately remains asymptomatic for a long period of time; therefore, most cases are diagnosed in advanced stages of the disease, when dissemination through peritoneal, hematogenous or lymphatic routes is already present [1]. However, a limited number of cases will be diagnosed in presumed early stages of the disease. However, up to 15% of these cases prove to have positive lymph nodes, which will significantly influence the long-term prognosis [2]; in the meantime, routine performance of extended pelvic and para-aortic lymph node dissection in presumed early stage ovarian cancer will lead to an unnecessary surgical procedure in up to 80% of cases who have otherwise negative lymph nodes [3,4,5,6]. Moreover, performing such procedures will increase the risk of developing perioperative complications, which might significantly influence the quality of life [7,8]. Therefore, identifying cases which present retroperitoneal para-aortic lymph node metastases will enable the oncologist to provide a better selection of cases that will benefit from adjuvant chemotherapy [2,3,9,10,11]. The aim of the current paper is to investigate the risk factors for developing para-aortic lymph node metastases in cases diagnosed with a presumed early stage of disease.

## 2. Materials and Methods

Data of patients submitted to surgery for presumed early stage ovarian cancer between 2014 and 2019 were retrospectively reviewed after receiving the approval of the Ethical Committee (11/January 2020). In all cases, the surgical procedure consisted of total hysterectomy en bloc with bilateral adnexectomy, random peritoneal biopsy, omentectomy, pelvic and para-aortic lymph node dissection, as well as peritoneal washing. Pelvic lymph node dissection consisted of removing the lymph node groups at the level of the common and external iliac vessels and obturatory fossa, while para-aortic lymph node dissection consisted of removing the lymph node groups situated in the close proximity of the abdominal aorta, inferior cava vein and in between the two vessels from the renal vessels to the aortic and caval bifurcation. All cases were classified according to the 2014 International Federation of Obstetrics and Gynecology classification (FIGO 2014) [12]. Statistically significant differences were considered if a *p*-value lower than 0.05 was obtained. In order to compare different parameters, Fischer’s exact test was used due to the relatively low number of cases introduced in the current study. 

## 3. Results

Between 2014 and 2019, 48 patients with presumed early stage ovarian cancer were submitted to surgery with curative intent, the median age at the time of surgery being 43.4 years (range = 28–56 years). According to their menopausal status, there were 13 postmenopausal women. The preoperative diagnostic was suspected based on the detection of higher levels of CA 125 (the median value being 330 U/mL) in association with the imaging detection of ovarian masses/cysts with uncertain aspect. In all cases, surgery consisted of total hysterectomy en bloc with bilateral adnexectomy, pelvic and para-aortic lymph node dissection, omentectomy, serial peritoneal biopsies and also an associated resection of all suspect lesions found at the level of the abdominal cavity and peritoneal washing. Intraoperative details are presented in Table 1.

In all cases, pelvic and para-aortic lymph node dissection was performed, the borders of the lymph node dissection being represented by the origin of the epigastric artery caudally (for the pelvic lymph node dissection) and, respectively, the origin of the renal artery cranially (for the para-aortic lymph node dissection). The median number of retrieved pelvic lymph nodes was 19 while the median number of the retrieved para-aortic lymph nodes was 14. Intraoperative and histopathological details of the lymph node dissection are presented in Table 2.

The median length of the surgical procedure was 130 min (range = 90–160 min), the median estimated blood loss was 350 mL (range = 100–550 mL), while the median length of the hospital stay was 6 days (range = 4–13 days). The histopathological studies confirmed the presence of positive pelvic lymph nodes in 18% of cases and, respectively, positive para-aortic lymph nodes in 22% of cases. However, all cases presenting positive pelvic nodes also had associated positive para-aortic lymph nodes. According to these findings, all cases with positive retroperitoneal lymph nodes were upgraded to FIGO stage III of disease and were therefore confined to the oncology department to be submitted to adjuvant chemotherapy. Patients with positive lymph nodes were further classified as IIIA1 (i) if the dimension of the metastatic deposits was lower than 10 mm (in three cases), IIIA1 (ii) if the dimension of the metastatic deposits was larger than 10 mm (in five cases) and IIIB if macroscopic, lower than 2 cm, extrapelvic peritoneal metastases were encountered (in the remaining three cases). None of these cases presented macroscopic peritoneal metastases larger than 2 cm; therefore, none of them were upstaged to FIGO stage IIIC of disease.

In order to determine the risk factors for developing lymph node metastases in apparently early stage ovarian cancer, we conducted an univariate analysis in which we studied the influence of age, menopausal status, initial FIGO stage at diagnosis, laterality of the tumor, histology and degree of differentiation on the risk of developing node metastases. The univariate analysis demonstrated that the presence of positivity of the retrieved lymph nodes was significantly associated with the serous histopathological subtype as well as with the degree of differentiation. Therefore, patients diagnosed with serous ovarian carcinoma had a significantly higher rate of positive lymph nodes (when compared to the other histopathological subtypes, *p* = 0.002). In the meantime, cases diagnosed with poorly differentiated tumors also exhibited a significantly higher rate of positive lymph nodes when compared to the other degrees of differentiation (*p* = 0.004). Surprisingly, neither the laterality of the tumor nor the presumed FIGO stage at diagnosis influenced the risk of developing such metastases. Data obtained at statistical analysis is presented in the table below (Table 3).

## 4. Discussion

The issue of lymph node metastases in presumed early stage ovarian cancer has a particular interest among surgical oncologists, gynecological oncologists and medical oncologists worldwide. It can be observed from the data presented so far that an important number of cases diagnosed with presumed early stage cancer already have, at the time of diagnosis, positive microscopic and even macroscopic lymph nodes, upstaging in this way the disease to a FIGO stage III malignancy. Therefore, all these cases, if submitted to standard treatment for early stage ovarian cancer, are at risk for developing early recurrent disease and a particularly poor long-term prognosis due to mis-staging. In the meantime, routine performance of extended lymph node dissection might predispose a significant number of cases to overtreatment and its secondary early-term and even long-term complications [2,4,13,14].

In order to increase the rates of preoperative detection of potential positive lymph nodes, certain authors proposed routine association of positron emission computed tomography. In the study conducted by Signorelli et al. published in 2013, the authors included 68 patients with presumed early stage ovarian cancer in which routine positron emission tomography, as well as systematic lymph node dissection, was performed. The authors underlined the fact that among the 12 cases who finally presented lymph node metastases at the histopathological studies, 10 cases had been correctly previously identified at the imaging studies. Therefore, the authors concluded that this imaging tool could be safely used in order to identify cases in which systematic lymph node dissection could be avoided, especially based on the high negative predictive value of the method [8]. Another promising method which might provide a more accurate identification of patients who present ovarian cancer lymph node metastases even in apparently early stages of the disease is represented by the sentinel node detection [4]. The method, which has been widely implemented in cases diagnosed with early stage breast cancer, melanoma and even gynecological cancers (such as endometrial cancer or cervical cancer), is still under evaluation in patients with presumed early stage ovarian cancer, further studies being still needed before introducing it as part of the standard therapeutic protocol [4,15,16,17,18].

One of the first studies, which designed a nomogram-based analysis in order to identify cases at risk of developing para-aortic lymph node metastases, was conducted by Bogani et al., on 290 patients with presumed early stage disease. According to their study, the authors demonstrated that bilateral lesions as well as high-grade serous histology represent the strongest predictors for para-aortic lymph node metastases even in cases with presumed early stage disease [2].

A similar conclusion was also presented by the study conducted by Zhou et al. [19]. In the paper published in 2016, the Chinese authors came to demonstrate that systematic lymph node dissection should be performed in cases diagnosed with poorly differentiated tumors, with serous histology and higher values of CA125 at the time of diagnosis. Therefore, cases in which the preoperative levels of CA125 surpass 740 U/mL seem to have a higher risk of associated para-aortic lymph node metastases [19].

An interesting study which investigated the effectiveness of surgical staging in cases with apparent early stage ovarian cancer has been recently published by Hengeveld in 2019. The study included all patients submitted to surgery with presumed early stage disease between 2005 and 2017 in Danish and Dutch hospitals [20]. Finally, there were 1234 cases that had been preoperatively presumed to be classified as FIGO stage I disease; in all cases, omentectomy, pelvic and para-aortic lymph node sampling or lymph node dissection, as well as multiple peritoneal biopsies, were retrieved. After analyzing the specimens, the histopathological studies revealed the fact that 20 patients were finally upstaged due to the presence of positive pelvic lymph nodes (in seven cases), positive para-aortic lymph nodes (in 12 cases) and both pelvic and para-aortic lymph nodes (in one case). Moreover, the authors underlined the fact that a total of 207 cases were upstaged after applying this protocol, with other sites of involvement being represented by the omentum, peritoneum or positive cytology. However, the authors underlined the fact that in another 50 cases, the malignant process was down-staged after applying this protocol, as the histopathological analysis of the macroscopically suspect lesions was not able to confirm the disease. This fact was rather explained by the absence of bilateral lesions and the absence of capsular invasion, respectively. Similarly to our study, the presence of serous histology as well as the poorer degree of differentiation significantly impacted on the risk of further upstaging. Other factors that were significantly associated with upstaging were represented by higher age, the postmenopausal status as well as the endometroid histology. Therefore, when it comes to the type of histology that is mainly associated with a poorer prognosis, according to this study, serous and endometroid histology versus any other type of tumor were associated with higher rates of upstaging. Moreover, the authors underlined the fact that upstaging was responsible for changing the plan of treatment in 35.1% of cases. Therefore, the importance of an adequate staging was underlined once again; in the meantime, the study came to demonstrate that a significant proportion of cases that were finally upstaged originated from cases with serous or endometrial histology in association with a lower degree of differentiation [20].

Another extremely interesting study that came to demonstrate the effect of the upstaging of presumed early stage ovarian cancer has been recently published in the *New England Journal of Medicine* in 2019. In this paper, the authors came to demonstrate that seven patients out of the 15 cases that were included in the presumed early stage ovarian cancer group presented in fact para-aortic lymph node metastases and were therefore upstaged to FIGO stage IIIC of disease. Moreover, the authors underlined the fact that performing para-aortic lymph node dissection in presumed early stages of the disease will probably prolong the surgical procedure (which is otherwise a short one) by almost an hour and will not predispose to such important complications when compared to cases diagnosed in advanced stages of the disease. In the meantime, routine association of this procedure in cases with advanced stages will also prolong a more demanding and laborious surgical procedure by another hour and will predispose to significant postoperative complications, such as a larger amount of ascites and lymphorrhea. Moreover, the authors also demonstrated that performing systematic lymph node dissection in patients with clinically negative lymph nodes increases the risk of perioperative complications without improving the long-term outcome [21]. 

## 5. Conclusions

Even in cases with presumed early stage ovarian cancer, a certain number of cases present lymph node metastases. Therefore, in such cases, the patients will be automatically upstaged and the therapeutic strategy will be modified. According to our study, the risk of developing para-aortic lymph node metastases seems to be significantly correlated with the serous histology as well as with a poorer degree of differentiation. Other factors that have been proven to modify this risk are represented by the FIGO stage, age at diagnosis and menopausal status. However, in our study none of these parameters seemed to significantly influence the risk of positive para-aortic lymph nodes. However, larger studies are still needed in order to provide a better identification of cases at risk of developing distant lymph node metastases. 

## Figures and Tables

**Table 1:** Preoperative and intraoperative characteristics of the 48 patients diagnosed with presumed early stage ovarian cancer.

Parameter	No. of Cases
Total number of patients	48
FIGO stage at diagnostic:	
I	23
II	25
Laterality of the tumors:	
Unilateral	19
Bilateral	29
Histopathological findings:	
Serous adenocarcinoma	23
Endometroid carcinoma	13
Clear cell carcinoma	10
Mucinous carcinoma	2
Degree of differentiation:	
Well differentiated	9
Moderately differentiated	29
Poorly differentiated	10

**Table 2:** Intraoperative and histopathological details of the lymph node dissection.

Parameter	Number
Number of retrieved pelvic lymph nodes (median)	19
Number of positive pelvic lymph nodes (median)	3
Number of retrieved para-aortic lymph nodes (median)	14
Number of positive para-aortic lymph nodes (median)	1
Number of cases with positive pelvic lymph nodes	9 of 48 cases
Number of cases with positive para-aortic lymph nodes	11 of 48 cases

**Table 3:** Analysis of risk factors for para-aortic lymph node metastases.

Risk Factor	Sample Number (%)	No. of Cases with Positive Para-Aortic Lymph Nodes	Hazard Ratio	95% Confidence Interval	*p*-Value
**Age:**			1.46	0.751–3.674	*p* = 0.389
<50 years	31 (65%)	8 (73%)			
>50 years	17 (35%)	3 (27%)			
**Histology:**			2.89	1.623–9.354	*p* = 0.002
Serous	23 (48%)	8 (73%)			
Other histopathological type	25 (52%)	3 (27%)			
**Menopausal status:**			0.65	0.522–4.138	*p* = 0.417
Premenopausal	35 (73%)	7 (64%)			
Postmenopausal	13 (27%)	4 (36%)			
**Initial FIGO stage at diagnostic:**			1.3	0.782–3.457	*p* = 0.276
I	23 (48%)	6 (54%)			
II	25 (52%)	5 (46%)			
**Laterality of the tumor:**			0.87	0.673–3.416	*p* = 0.424
Unilateral	19 (39%)	4 (36%)			
Bilateral	29 (61%)	7 (64%)			
**Degree of differentiation:**			0.433	1.773–10.157	*p* = 0.004
Well differentiated	9 (19%)	1 (9%)			
Moderately or poorly differentiated	39 (81%)	10 (91%)			
